# Accessibility and usability OCW data: The UTPL OCW

**DOI:** 10.1016/j.dib.2017.06.007

**Published:** 2017-06-15

**Authors:** Germania Rodríguez, Jennifer Perez, Samanta Cueva, Rommel Torres

**Affiliations:** a Universidad Técnica Particular de Loja, Ecuador; b Universidad Politécnica de Madrid, Spain

**Keywords:** Accessibility questionnaire, Usability questionnaire, Accessibility criteria, Usability criteria, OCW UTPL, Accessibility problems and solutions, Usability problems and solutions

## Abstract

This data article provides a data description on article entitled “A framework for improving web accessibility and usability of Open Course Ware sites” [3] This Data in Brief presents the data obtained from the accessibility and usability evaluation of the UTPL OCW. The data obtained from the framework evaluation consists of the manual evaluation of the standards criteria and the automatic evaluation of the tools Google PageSpeed and Google Analytics. In addition, this article presents the synthetized tables from standards that are used by the framework to evaluate the accessibility and usability of OCW, and the questionnaires required to extract the data. As a result, the article also provides the data required to reproduce the evaluation of other OCW.

**Specifications Table**

**Table:** 

Subject area	*Computer Science and Education*
More specific subject area	*Software Engineering and Open Educational Resources*
Type of data	*Tables*
How data was acquired	*Questionnaire and software tools*
Data format	*Analyzed.*
Experimental factors	*The results are summarized and concluded from the results of questionnaires*
Experimental features	*Manual and Automatic Evaluation was performed by observers and subject using tools and questionnaires*
Data source location	*Universidad Técnica Particular de Loja*
	*City: Loja*
	*Country: Ecuador*
	*and/or Latitude & Longitude (& GPS coordinates) for collected samples/data if applicable*
Data accessibility	*The data is with this article*

**Value of the data**

• The evaluation of usability and accessibility criteria using the framework presents problems and solutions. These problems may be undergone by other OCW, and their solutions may be applied to those OCW.
• The data obtained from the usability evaluation of the UTPL OCW using the tools Google PageSpeed and Google Analytics are a source of data to other research works of the area, since these data can be used by them for new analysis and comparisons.
• The questionnaire and synthetized tables from standards, which are used by the framework to evaluate the accessibility and usability of OCW, can be applied to evaluate the accessibility and usability of other OCW.

## 1 Data

Table S1 presents the results of the accessibility criteria evaluation of the OCW UTPL case study (See Appendix A) describe on [3]. Specifically, the evaluation of the accessibility criteria is presented in terms of the principles, criteria and accessibility level (A, AA, AAA) conforming to the Web Content Accessibility Guidelines WCAG 2.0 [5]. In Table S1, for each criterion in which problems were found in the UTPL OCW evaluation, it specifies the identified problems and their improvements, as well as the accessibility level resulting from the evaluation being:

• **Level A** (lowest)**:** The web page satisfies all the Level A Success Criteria, or conformance to an alternate version is provided.
• **Level AA** (medium)**:** The web page satisfies all the Level A and Level AA Success Criteria, or a Level AA alternate version is provided.
• **Level AAA** (highest)**:** The web page satisfies all the Level A, Level AA and Level AAA Success Criteria, or a Level AAA alternate version is provided.

With regard to usability evaluation, Table S2 presents the data resulting from the usability criteria evaluation of the UTPL OCW (See Appendix A). This table is organized by following the usability guide of the standard ISO 9142-11. This usability guide considers the degree of the three usability measures: effectiveness, efficiency and satisfaction. Therefore, the table presents the identified problems/errors and their possible solutions in terms of effectiveness, efficiency and satisfaction; and it also presents results of the aspects, criteria and priority in the UTPL OCW educational/training website following the Sirius framework [4].

On the other hand, Table 1, Table 2 present the results obtained from the automatic usability evaluation of the UTPL OCW using tools for this purpose. Specifically, Table 1 shows its usability results in terms of the priority, the identified errors and the suggested solution by the Google PageSpeed tool [2]. Google PageSpeed measures the performance and the loading speed of a website accessed from mobile and desktop devices. From the analysis of the website, the tool also provides a set of tips for improving the usability.

**Table 1:** Results from the tool Google PageSpeed.

**Priority**	**Error description**	**Suggested solution**
*Load speed*		PC׳s: 68/100
		Mobiles: 56/100
*Very high*	This rule is triggered when Google PageSpeed detects that the response of the user׳s server does not include explicit cache headers or specifies that the resources are stored in cache for a short time.	Avoid problems in the browser׳s cache storage
	This rule is triggered when the tool detects that those resources of the page that can be compressed have not been processed with HTTP compression.	Enable compression
*High*	This rule is triggered when Google PageSpeed detects that the HTML code makes reference to an external JavaScript file that blocks the display of content in the upper half of the page.	Delete the processing of JavaScript and CSS blocking in the content of the first displaying screenshot
	This warning occurs when the size of one of the resources could be reduced through minimization. The resource minimization refers to deleting unnecessary bytes such as extra spaces, line breaks and indents. Minimizing HTML, CSS and JavaScript codes, it is possible to accelerate downloading, analysis and execution times.	Minimize CSS
	This rule is triggered when Google PageSpeed detects that the server׳s response time is greater than 200 Ms. The server׳s response time indicates the time taken for loading the HTML code needed to display the page from the server, subtracting the latency of the network between the server and Google. There may be differences between one loading and another, but they should not be very different. In fact, a highly variable server response time could indicate an underlying performance problem.	Reduce the response time of the server
	This rule is triggered when Google PageSpeed detects that the images of a page may be optimized without affecting their visual quality by reducing the size.	Optimize images
	This rule is triggered when Google PageSpeed detects that the size of one of the resources can be reduced through minimization. The minimization of resources refers to the deletion of unnecessary bytes such as extra spaces, line breaks and indents.	Minimize HTML
	This rule is triggered when Google PageSpeed detects that the size of one of the resources can be reduced through minimization. The minimization of resources refers to the deletion of unnecessary bytes such as extra spaces, line breaks and indents. Minimizing HTML, CSS and JavaScript codes makes it possible to accelerate downloading, analysis and execution times.	Minimize JavaScript

**Table 2:** Results from the Google Analytics tool.

*Interval of time*	2010–2015	
*Number of visits*	117,433	
**Criterion**	**Item**	**Percentage**

*Countries*	Ecuador	32.12%
	Peru	10.89%
	Russia	6,44%
	United Kingdom	6.17%
	Not set	4.76%
	Mexico	4.72%
	Colombia	4,13%
	No significant values (less than 3%)	25,64%
*Browser*	Chrome	73.59%
	Firefox	13.88%
	Safari	4.22%
	No significant values (less than 3%)	8.31%
*Operating system*	Windows	63,97%
	Mac OS	22.41%
	Android	4.26%
	Not set	3,09%
	iOS	3.04%
	Linux	3,04%
	No significant values (less than 3%)	0,49%
*Mobile devices*	Apple	40,37%
	Samsung	22,89%
	Not Set	9,64%
	Intel	4,82%
	Alcatel	3,01%
	LG	3,01%
	Sony	3,01%
	No significant values (less than 3%)	16.26%
*Mobile operating system*	Android	56,63%
	iOS	40.36%
	Windows Phone	2,94%
	Windows	0,60%

Table 2 shows the results obtained from the automatic evaluation of Google Analytics [1] of the UTPL OCW. Google Analytics provides detailed reports and statistical information about the visits to a website, as well as the browser or the type of device that is connected to. The results are presented in Table 2 in terms of the countries, browsers, operating systems, mobile devices and mobile operating system used in the connection to the UTPL OCW website.

## 2 Experimental design, materials and methods

To evaluate the accessibility and usability of the UTPL OCW site, the standards were synthesized into tables and they were adapted to OCW requirements. Table S5 synthesizes the WCAG 2.0 guidelines [5] to evaluate the accessibility of OCW websites and to suggest improvements for each criterion (See Appendix A).

On the other hand, the usability criteria have been selected from Sirius framework [4]. This framework is one of the most complete proposals based on heuristics to measure usability, and by extension, *satisfaction*. This framework measures 83 criteria of 10 aspects under evaluation in a quantitative and qualitative way. These aspects are: General Aspects (GA), Identify and Information (II), Structure and Navigation (SN), Labelling (LB), Layout of the Page (LY), Comprehension and easy Interaction (CI), Control and Feedback (CF), Multimedia Elements (ME), Search Elements (SE), and Help Elements (HE). Each aspect is composed of a set of measurable criterion synthesized in Table S6 (See Appendix A), and the numerical measures and its corresponding category (label value) are presented in Table 3.

**Table 3:** Sirius evaluation values for a usability criteria.

**Label value**	**Definition**	**Numerical value**
NWS	Not compliant in the whole site	0
NML	Not compliant in the mail links	2,5
NHP	Not upheld in the home page	5
NSP	Not compliant in one or more subpages	7,5
YES	Fully compliant	10
NA	Criterion not applicable in the site	–

A questionnaire was provided to the subjects that evaluate the accessibility and usability criteria. They are presented in Tables S8 and S9 of the Appendix A.
