# Supplementary material for: Accessibility and usability OCW data: The UTPL OCW
Source: Data Brief. 2017 Jun 15;13:582–6. doi: 10.1016/j.dib.2017.06.007 (PMC5496481; doi:10.1016/j.dib.2017.06.007)
Supplement: Supplementary file 3 — Supplementary material [file mmc3.pdf]

Table 2. Proposal for the improvement of identified usability problems

Usability Measure ISO 9142-11	Aspects	Criterion	Priority in an educational / training website	Possible problem / error	Solution
**EFFICIENCY**	Does not apply.	Does not apply.	Does not apply.	Excessive cache storage in the browser.	Establish values for the attributes **expiry date** or **maximum age** in the HTTP headers for static resources such as images that have been downloaded in previous visits, stored in cache.
	Does not apply.	Does not apply.	Does not apply.	Compression is disabled.	Adjusting the compression of resources using GZIP may reduce the number of bytes sent via the network. It is suggested to enable compression of the following resources to reduce their transfer size by 131.4KB (67% reduction).
**EFFICACY**	Does not apply.	Does not apply.	Does not apply.	Website loading errors in web browsers.	Carry out functionality tests on all browsers selected by the evaluated users.
	Does not apply.	Does not apply.	Does not apply.	Website loading errors in mobile devices.	For subsequent studies, it is proposed changing the interface of the UTPL OCW to be browsed exclusively on mobile devices.
**SATISFACTION**	**General Aspects (GA)**	GA1: Concrete and well–defined goals of the website.	MA	The site objectives not being stated explicitly in the OCW website.	Place the objectives of the OCW website explicitly on a section that is accessible to the user.
		GA2: Precise and complete content and services offered.	CR	The available services are not easily identifiable in the OCW website.	The OCW website services are clearly identified.
		GA3: User–oriented general structure of the website.	MA	The general structure of the OCW website is not understandable to the user.	Maintain a structure that is easy to understand.

		GA6: Coherent general design of the website.	MA	The design of the OCW website is not coherent throughout its pages.	Maintain a structure design that is coherent and easy to understand among that pages that are part of the OCW website.
		GA7: The user's language is used.	MA	The language defined for the OCW not being that required by the user, or not having the option to adjust it.	Provide the option to select the language in which the OCW website content is presented to the user.
	**Information Identity (Ii)**	II7: Information is offered about the author, sources and creation and revision dates of the document for articles, news, and reports.	MA	Not placing information, such as the author, creation and modification date of the OCW website content.	Place information such as the author, creation and modification date of the content in the OCW website.
	**Structure and navigation (SN)**:	SN2: Adequate organization and browsing structures.	MA	Inadequate hierarchical organization of the content and browsing indices in the OCW website.	Establish an organization and browsing structure (index) that is understandable by the user of the OCW website
		SN4: Control over the number of elements and terms per element in the browsing menus.	MA		
		SN5: Balance between depth and width in the case of hierarchical structure.	MA		
		SN6: Easily–recognizable links.	MA	Links are not in a unique and recognizable format in the OCW website.	Ensure that the links have a unique and recognizable format in the OCW website.
		SN7: Characterization of the link that indicates their states (visited, active).	MA	The lack of a characterization of the visited or active links in the OCW website.	Ensure that the links have a different format for active or visited pages in the OCW website.

		SN8: Link redundancy has been avoided.	MA	Presence of broken links in the OCW website.	Eliminate broken links in the OCW website.
		SN9: The existence of broken links has been avoided.	MA		
		SN13: There are browsing elements that orient the user about where they are and how to trace back their navigation (breadcrumbs, colored tabs)	MA	The lack of elements that inform the user of their location in the OCW website.	Provide elements or mechanisms that orient the user about their location in the OCW website.
	**Layaout of the page (LY):**	LY8: The printed version of the page is correct.	MA	The printed version of the OCW website content is not easily legible.	Maintain the adequate structure, content and formats for the OCW website printed version.
		LY9: The page text is read without difficulty.	MA	The OCW website text content is not easily legible.	Maintain the adequate structure, content and formats to facilitate reading of the OCW website.
	**Comprehen sibility and ease of Interaction (CI)**	CI1: A clear and concise language is employed.	MA	The language used on the OCW website is excessively technical or complex, complicating its reading.	Employ an adequate, clear and concise language in the OCW website.
	**Multimedia elements (ME)**:	ME2: Understandable pictures.	MA	The figures or pictures in the OCW website are not legible or understandable.	Ensure that the format and size of the figures and pictures of the OCW website are adequate to be legible and understandable.
	Search (SE)	SE1: If necessary, it is accessible from all website pages.	MA	The search option for the OCW website is not accessible from all OCW website pages.	Ensure that the OCW website search option is accessible from all pages of the website.

		SE4: The text box is wide enough.	MA	The text box size for searching the OCW website is inadequate.	Ensure that the box size for searching the OCW website is adequate.
		SE5: Simple and clear search system.	CR	The OCW website search engine is not simple and clear for the user.	Ensure that the search engine of the OCW website is simple and clear for the user.
		SE7: Showing the search results in a way that is understandable for the user.	MA	The results of a search carried out on the OCW website is not understandable for the user.	Ensure that the results provided by a search carried out in the OCW website are structured and have an adequate format that facilitates the user's understanding.
		SE8: Assisting the user in the event that results cannot be provided for a given query.	MA	Assistance mechanisms are not provided for the users of the OCW website in the event of a search.	Provide assistance mechanisms to the OCW website users in the event of a search.
	**Help (HE**)	HE2: Ease of access to and return from the help system.	ME	Lack of help.	Introduce a user manual with simple and understandable information.<br>Have a customized and reality–oriented list of FAQs for the UTPL OCW.
