# Supplementary material for: Accessibility and usability OCW data: The UTPL OCW
Source: Data Brief. 2017 Jun 15;13:582–6. doi: 10.1016/j.dib.2017.06.007 (PMC5496481; doi:10.1016/j.dib.2017.06.007)
Supplement: Supplementary file 4 — Supplementary material [file mmc4.pdf]

Table5: Guide for the accessibility criteria, problems and improvements to be considered for OCW websites.

PRINCIPLE / WCAG 2.0 accessibility guidelines	WCAG 2.0 accessibility criterion	Accessibility level	Possible problem / error	Solution / improvement
**PERCEPTIBLE:**<br>The information and components of the user interface must be presented to the user in such a way that they can perceive them.				
**1.1 Text alternatives:** Provide text alternatives for any non–textual content, such as large font, Braille, language, symbols, or simplified language.	1.1.1 Non–textual content: Any non–textual content that is presented should feature alternative text with an equivalent meaning.	**A**	Non–textual content without alternative text within the OCW website content.	Include alternative text in all non–textual content<br>Validate the OCW website using automatic accessibility evaluation tools.
**1.2 Based on mean time**: Provide alternatives for time–based means of communication.	1.2.1 Audio–only and video–only (pre–recorded): Provide pre–recorded audio and video.	**A**		
	1.2.2 Subtitles (pre–recorded): Provided for all audio present in the content, except when the audio is alternative and is identified as such.	**A**		
	1.2.3 Automatic description of alternative media (pre–recorded): Must be provided for all content, except when it is alternative and is identified as such.	**A**		

	1.2.4 Subtitles: Subtitles are provided for all audio or synchronous multimedia content.	**AA**	OCW website content without titles or subtitles.	Include titles or subtitles in all OCW website content.
	1.2.5 Audio description: Must be included in all pre–recorded videos in the multimedia content.	**AA**	OCW videos without audio description.	Include audio description for videos that are part of the OCW website.
	1.2.6 Sign language (pre–recorded): An interpretation of the content in sign language must be provided for all pre–recorded audio in multimedia content.	**AAA**	OCW website content without a pre–recorded description in sign language.	Include a pre–recorded sign–language description to the OCW website content.
	1.2.7 Extended audio description (pre–recorded): Where there is a pause in the multimedia content, an extended audio description must be included in all pre–recorded videos in multimedia content.	**AAA**	OCW website videos without extended audio description.	Include extended audio description for videos that are part of the OCW website.
	1.2.8 Multimedia alternative (pre–recorded): A descriptive transcription must be provided for all pre–recorded media.	**AAA**	Pre–recorded content in the OCW website without alternative multimedia.	Include alternative multimedia for pre–recorded content of the OCW website.
	1.2.9 Audio only: A descriptive transcription is offered for all direct content that contains only audio.	**AAA**	Audio content in the OCW website without content transcription.	Include a transcription of the OCW website audio content.
**1.3 Adaptable:** Create content that can be presented in a	1.3.1 Information and relations: The	**A**	Non–customizable information of structure and relations in the OCW	Give the option to make adjustments through programming

variety of ways without losing information or structure	presented information, structure and relations are programmable or are available in text format.		website	of the OCW website navigation structure.
	1.3.2 Meaningful sequence: When the sequence of the presentation affects the meaning, the correct sequence can be determined through programming.	**A**	Meaningless sequence of OCW content.	Provide a meaningful presentation sequence and navigation.
	1.3.3 Sensory characteristics: The instructions provided for the understanding and the content are not based solely on sensory aspects, such as shape, size, visual location, orientation or sound.	**A**	OCW website content centered on aesthetics instead of the content that is being transmitted.	Validate that the OCW website content be centered mainly on its purpose, rather than its aesthetics.
**1.4 Distinguishable:** Make it easy for the users to see and listen to content, including the separation from the main background plane.	1.4.1 Use of color: Color must not be the only visual means for information transmission.	**A**	Color used excessively or for different purposes in the OCW website.	Use color in moderation and for specific purposes.
	1.4.2 Audio control: Any audio that is reproduced for longer than 3 seconds must have the option to be paused or disabled.	**A**	Audio included in the OCW websites without providing the user with control mechanisms.	Provide the user with control over the audio present in OCW websites.
	1.4.3 Contrast: The visual presentation of text and text images have a contrast ration of at least 4.5: 1, with the exception of large text, incidental text or banners.	**AA**	Colored content or images with contrast lower than the established minimum of 4.5:1.	Use on–line tools to validate that the color contrast is adequate.

	1.4.4 Change the font size: Give the option of changing the font size, except for titles and text images.	**AA**	Text content in the OCW website without the option to adjust it.	Give the option to adjust the text content in the OCW website.
	1.4.5 Text images: Only in the case of customizable images or particular text presentations.	**AA**	Use of text images for different purposes than those accepted.	Eliminate text images where they are not necessary.Add an attribute “alt” to each image in the website.
	1.4.6 Contrast (improved): The visual presentation of text and images must have a contrast ration of 7:1, with the exception of large text, incidental text or banners.	**AAA**	Content or colored images with a lower contrast than the established minimum 4.5:1.	Include content and images using foreground colors with a contrast ratio equal or greater than 7:1.
	1.4.7 No audio background or with low volume: Pre–recorded audio content is allowed if (1) it is foreground speech, (2) it is CAPTCHA or a banner; and (3) it does not have a musical purpose.	**AAA**	Audio content in the OCW website for a purpose other than what is allowed: (1) speech or (2) CAPTCHA.	Eliminate audio from the OCW website that is present for any purpose other than the allowed: (1) speech or (2) CAPTCHA.
	1.4.8 Visual presentation: Understandable visual content, customizable foreground colors, no more than 80 characters per line, justified text, line spacing, font size that can be increased to 200% its original size.	**AAA**	OCW site content that is difficult to understand because of its format.	Adjust the content format and visual presentation to the established norms: a maximum of 80 characters per line, justified text, line spacing, with a font size adjustable up to 200% of its original size.
	1.4.9 Text images (no exception): They should not be employed as mere decoration. They must transmit essential information.	**AAA**	Text or decorative images without identification (title attribute) or purpose in the OCW website.	Identify and validate the purpose of the decorative or text images that are part of the website.

OPERABLE

The user interface and navigation must be operable.

**2.1 Keyboard Access**: Make every functionality available through the keyboard.	2.1.1 Keyboard: Every functionality must be operable though a keyboard interface.	**A**	Functionality of content in the OCW website without keyboard access. Content components that can be accessed but not exited using the keyboard.	Ensure that all content blocks and functionalities must be accessible to the keyboard.
	2.1.2 No keyboard traps: If the keyboard can move onto a component in the page, its focus should also be able to exit that component through the keyboard.	**A**		
	2.1.3 Keyboard (no exception): Every content functionality can be operated through a keyboard interface, without specific timing of individual keys.	**AAA**		
**2.2 Enough Time**: Provide the users enough time to read and use the content.	2.2.1 Adjustable time: For each established time limit in the content.	**A**	An OCW website having established times for its content or functionalities that cannot be adjusted by the user.	Give the user the option to adjust the established times for the content and functionalities of the OCW website, if any.
	2.2.2 Pause, stop, hide: For all information that is moving, blinking, being displaced or automatically refreshed.	**A**	An OCW website containing information that moves, blinks, is displaced or refreshed automatically, without the option of being controlled by the user.	Give the user the option to control the information that moves, blinks, is displaced or refreshed automatically in the OCW website.
	2.2.3 No timing: The content and functionality must not have time limits, except for multimedia content.	**AAA**	An OCW website having established times for their content or functionalities.	Eliminating limited times for content or functionalities in the OCW website.

	2.2.4 Interruptions: Can be postponed or cancelled by the user, with the exception of emergencies.	**AAA**	An OCW website that contains interruptions that are not urgent.	Eliminating any type of interruptions, except for emergencies.
	2.2.5 Re-authentication: The user should be able to re–authenticate their identity and continue their activities without losing any information of the current page if their session expires.	**AAA**	The absence of a re–authentication option for the user.	Give the user a re–authentication option.
**2.3 Seizures**: The content should not be designed in a way that may cause seizures.	2.3.1 Three flashes or below threshold: The websites must not contain anything that flashes more than three times in any given one second period or with the flash being under the general flash and red flash thresholds.	**A**	An OCW website that contains content with more than three flashes per second.	Eliminate content with flashes.
	2.3.2 Three flashes: The websites do not contain anything that flashes more than three times in any given second.	**AAA**		
**2.4 Navigable**: Provide the users help media for navigation, finding content and determining their location within the website.	2.4.1 Bypass blocks: A mechanism that allows jumping between content blocks that are repeated in multiple pages.	**A**	An OCW website that lacks mechanisms for moving between content blocks.	Provide the OCW website with components such as identification and location of the pages to maintain meaning and functionality for the user at any given time during navigation.
	2.4.2 Titled page: Web pages must have titles that describe their topic or purpose.	**A**	Pages and sub–pages of the OCW website that are not appropriately identified with the title attribute.	
	2.4.3 Focus order: If a web page can	**A**	Lack of mechanisms to show the	

	be browsed sequentially and the navigation sequence affects its meaning or functionality, there must exist components that maintain the meaning and functionality.		user's location at any given moment.	
	2.4.4 Purpose of a link: The purpose of every link must be identified by its text.	**A**	The links text in the website are not sufficiently descriptive.	Ensure that the text of the OCW website links be sufficiently descriptive and do not repeat.
	2.4.5 Multiple media: There are multiple ways to locate a web page within a collection of webpages.	**A**		
	2.4.6 Titles and labels: Titles and labels must describe the topic or purpose appropriately.	**AA**	Titles and labels in the OCW website are not descriptive or appropriate.	Ensure that the titles and labels of the OCW website are descriptive and appropriate.
	2.4.7 Visible focus: Any user interface that can be operated with the keyboard must have a visible indicator of the keyboard focus.	**AA**	The lack of a visible focus of the user location in the content or functionality of the OCW.	Provide XX? to the user with a visible focus on their location within the content and functionality of the OCW website.
	2.4.8 Location: The user is provided with information about their location inside a collection of webpages.	**AAA**	No mechanism exists to show the user's location inside the OCW website.	Provide a mechanism that shows the user's location within the OCW website.
	2.4.9 Purpose of a link (unique link): There must not exist links with the same text linking to different locations.	**AAA**	The existence of links with the same text linking with different pages inside the OCW website.	Eliminate links with the same text that link to different pages within the OCW website.
	2.4.10 Section titles: Employed to organize the content.	**AAA**	Webpage titles that do not have the purpose of organizing the content.	Provide descriptive titles for the pages of the OCW website that

				allow organizing the content.
**UNDERSTANDABLE**<br>The information and functionality of the user interface must be intelligible.				
**3.1 Legible**: The provided content is legible and understandable.	3.1.1 Page language: The language of each page must be programmable or determined by the user.	**A**	Language of the OCW website that is not defined or not adjustable by the user.	Define the language of the OCW website, or providing a mechanism for the user to define it.
	3.1.2 Language in different parts: The language of each passage or phrase in the content can be selected, with the exception of names, technical terms or undetermined languages.	**AA**	Language of the OCW website that is not defined or not adjustable by the user.	Define the language of the OCW website, and providing a mechanism for the user to define it.
	3.1.3 Unusual words: Must be defined through adjacent text, a list of definitions, or a glossary.	**AAA**	The presence of abbreviations or technical terms in the OCW website that are not defined in a glossary.	Include a glossary of technical terms and abbreviations that are used in the OCW website.
	3.1.4 Abbreviations: A mechanism for expanding the meaning of abbreviations must be available.	**AAA**		
	3.1.5 Reading level: Whenever the text requires a more advanced reading level than secondary education, a version that does not require more advanced reading skills must be available.	**AAA**	The presence of excessively technical content or content that requires a very advanced reading level within the OCW website, without the option of a version with lower difficulty.	Provide a low–difficulty version for excessively technical content or content requiring an advanced reading level in the OCW website.
	3.1.6 Pronunciation: A specific mechanism must be provided to	**AAA**	The presence in the OCW website of ambiguous words, without the	Provide a specific mechanism to identify the meaning of words that

	identify the meanings of words that are ambiguous if their pronunciation is not known.		option to clarify their meaning or pronunciation in their context.	are ambiguous when their pronunciation is unknown.
**3.2 Predictable**: Present webpages and their operation predictably.	3.2.1 With focus: The context must not change when any component is focused on.	**A**	Context or interface changes in the OCW website triggered by changing the focus or by data input.	Ensure that there are no context or interface changes when focus is shifted or when data are input by the user.
	3.2.2 At entry: When any component of the user interface is changed, this does not automatically cause a context change.	**A**		
	3.2.3 Consistent navigation: The browsing mechanisms are repeated in different websites in the same order.	**AA**	Different browsing mechanisms for different pages of the OCW website.	Provide equal or similar browsing mechanisms in all pages of the OCW website.
	3.2.4 Consistent identification: Components that have the same functionality within a set of webpages are identified in a coherent manner.	**AA**	Contents or functionalities with the same purpose that are identified differently.	Ensure that content and functionalities with the same purpose are similarly identified.
	3.2.5 Changes by request: Context changes must be initiated by user request, and an option should be given to disable such changes.	**AAA**	Unsolicited context changes in the OCW website without the option to disable by the user.	Provide the option to change the context of the OCW website, as well as the option to disable these context changes.
**3.3 Input assistance**: Aid the users in avoiding and correcting errors.	3.3.1 Error identification: If a data input error is detected, the item with the	**A**	The lack of alerts indicating that user input is required, the lack of data validation, or suggestions to	Provide control, feedback and validation for the data input by the user in the OCW website.

	error must be automatically identified and described to the user through text.		correct possible errors.	
	3.3.2 Instructions or labels: Provided when the content requires the user's intervention.	**A**		
	3.3.3 Suggestions after the error: If an input error is detected, the appropriate suggestions are provided for its correction.	**AA**		
	3.3.4 Error prevention: In law, financial and similar websites, control options must be provided to revert, verify or confirm.	**AA**		
	3.3.5 Help: Context–sensitive help is provided.	**AAA**	Absence of help to the user in webpages and functionalities of the OCW website.	Provide a help mechanism for the user in webpages and functionalities of the OCW website.
	3.3.6 Error prevention: In websites where the user sends information, options must be provided to revert, verify and confirm.	**AAA**	The lack of controls in input formularies within the OCW website to revert, verify or confirm the data input.	Allow the users to control, give feedback and validate the data they input to the OCW website.

ROBUST

The content must be sufficiently robust to be reliably interpreted by a wide variety of user applications.

**4.1 Compatible**: maximize compatibility with the current and future user agents, including technical help.	4.1.1 On analysis: The elements have full open and close labels, correctly nested, without duplicated attributes.	**A**	The existence in the OCW website code of errors such as incomplete labels, undefined or ambiguous nesting, duplicated attributes, etc.	Eliminate errors such as incomplete labels, undefined or ambiguous nesting, duplicated attributes, etc. from the OCW website code
	4.1.2 Name, role, value: For each user interface component, the name and role can be selected. Their states, properties and values can be established by the user.	**A**	User interface components in the OCW website that lack name, role, state, properties, or established values.	Appropriately identify the user interface of the OCW website, including name, role, state, properties and values.
