# Supplementary material for: Accessibility and usability OCW data: The UTPL OCW
Source: Data Brief. 2017 Jun 15;13:582–6. doi: 10.1016/j.dib.2017.06.007 (PMC5496481; doi:10.1016/j.dib.2017.06.007)
Supplement: Supplementary file 5 — Supplementary material [file mmc5.pdf]

Table 6: Description of the aspects defined by Sirius for evaluating the usability of web sites.

ASPECTS	DESCRIPTION	Criteria
**General Aspects (GA)**	Elements related to the goals of the web site, the look and feel, the consistency and the updating level of contents.	GA1: Goals of the site are concrete and well defined.<br>GA2: Contents and services are precise and complete.<br>GA3: General structure of the site is user-oriented.<br>GA4: General look and feel is aligned to the goals of the web site.<br>GA5: General design of the web site is recognizable.<br>GA6: General design of the web site is coherent.<br>GA7: User's language is used.<br>GA8: Other languages are supported.<br>GA9: Translation of the page is complete and correct.<br>GA10: Web site is updated regularly.
**Identity and Information (II)**	Elements related to the identity of the site, the information provided about the supplier and the content authorship.	II.1 Identity or logo is significant, identifiable and visible.<br>II.2 Identity of the web site is present on every page.<br>II.3 Slogan or tagline is suited to the goal of the site.<br>II.4 Information about the web site or company is provided<br>II.5 Contact mechanisms are provided.<br>II.6 Information about privacy of personal data and copyright of web contents is provided.<br>II.7 Information about authorship, sources, creation and revision dates
**Structure and navigation (SN)**	Elements related to the suitability of the information architecture and the web site navigation.	SN.1 Welcome screen is avoided<br>SN.2 Structure and navigation are adequate.<br>SN.3 Element organization is consistent with conventions.<br>SN.4 Number of elements and terms per<br>element is controlled in navigation menus.<br>SN.5 Depth and breadth are balanced in the case of hierarchical structure.<br>SN.6 Links are easily recognized as such.<br>SN.7 Link depiction indicates its state (visited, active).<br>SN.8 Redundant links are avoided.<br>SN.9 Broken links are avoided.<br>SN.10 Self links to the current page are avoided.<br>SN.11 Image links indicate the content to be accessed.<br>SN.12 A link to the home page is always<br>present.<br>SN.13 Elements hinting where the user is and how to undo the navigation (breadcrumbs, coloured tabs) exist.<br>SN.14 A map of the site to directly access<br>contents without navigation exists.
**Labelled (LB)**	Elements related to the significance, correctness and familiarity of the labelled of the contents.	LB.1 Labels are significant.<br>LB.2 Labelling system is precise and consistent.<br>LB.3 Page titles are planned and correct.<br>LB.4 Home page URL is correct, clear, and easy to remember.<br>LB.5 Inner page URLs are clear.<br>LB.6 Inner page URLs are permanent.

**Layout of the page (LY)**	Elements related to the distribution and the appearance of the browsing and interface elements.	LA.1 Higher visual hierarchy areas of the page are used for relevant content.<br>LA.2 Information overload is avoided.<br>LA.3 Clean interface with no visual noise.<br>LA.4 White areas between information objects are provided for visual rest.<br>LA.5 Visual space on the page is used correctly.<br>LA.6 Visual hierarchy is correctly used to<br>express "part of" relationships between page elements.<br>LA.7 Page length is under control.<br>LA.8 Print version of the page is correct.<br>LA.9 Page text can be read easily.<br>LA.10 Blinking/moving text is avoided.
**Comprehensibility and ease of Interaction (CI)**	Elements related to the adequacy and quality of textual contents, icons and interface controls.	CI.1 Concise and clear language is used.<br>CI.2 Language is user friendly.<br>CI.3 Each paragraph expresses an idea.<br>CI.4 Interface controls are used consistently.<br>CI.5 Visible metaphors are recognizable and comprehensible by any user (e.g. icons).<br>CI.6 Coherent or alphabetic order in<br>drop-down menus.<br>CI.7 Available options in a user-input field can be selected instead of written.
**Control and feedback (CF)**	Elements related to freedom of the user navigation and the information provided to the user during his/her interaction process with the site.	CF.1 User controls the whole interface.<br>CF.2 User is informed about what is happening.<br>CF.3 User is informed about what has<br>happened.<br>CF.4 Validation systems are in place to avoid errors before the user sends information.<br>CF.5 Clear and non-alarmist information and recovery actions are provided to the user when an error has occurred.<br>CF.6 Response time is under control.<br>CF.7 Web site windows cancelling or<br>superimposing over browser windows are avoided.<br>CF.8 Proliferation of windows is avoided.<br>CF.9 User downloading of additional plugins is avoided.<br>CF.10 In task with several steps, user is<br>informed of the current step and the number of steps remaining to complete the task.
**Multimedia elements (ME)**	Elements related to the adequacy degree of the multimedia content presented in the web site.	ME.1 Images are well-cropped.<br>ME.2 Images are comprehensible.<br>ME.3 Images have the correct resolution.<br>ME.4 Some added value is provided by using images or animations.<br>ME.5 Cyclical animations are avoided.<br>ME.6 Some added value is provided by using sound.
**Search (SE)**	Elements related to the search engine implemented on web site.	SE.1 Accessible in every page if necessary.<br>SE.2 Easily recognizable.<br>SE.3 Easily accessible.

		SE.4 Text box width is enough.<br>SE.5 Simple and clear search system.<br>SE.6 Advanced search is provided.<br>SE.7 Search results are comprehensible for the user.<br>SE.8 User is assisted in case of empty results for a given query
**Help (HE)**	Elements related to the help offered to the user during the navigation through the site.	HE.1 Help link is located in a visible and<br>standard place.<br>HE.2 Easy access to and return from the help system.<br>HE.3 Context help is offered for complex tasks.<br>HE.4 FAQ query selection and redaction is correct.<br>HE.5 FAQ answers are correct.
