# Supplementary material for: Accessibility and usability OCW data: The UTPL OCW
Source: Data Brief. 2017 Jun 15;13:582–6. doi: 10.1016/j.dib.2017.06.007 (PMC5496481; doi:10.1016/j.dib.2017.06.007)
Supplement: Supplementary file 6 — Supplementary material [file mmc6.pdf]

Table 9: Questionnaire for accessibility evaluation

Based on Web Content Accessibility Guidelines 2.0

Evaluator:
Date: **Age:**
Type of Site: Educational / Training Sex: ☐ M ☐ F
URL:
Browser that will be evaluated:
Version of the browser

Instructions:

1.- For each of the following statements, check the box that best describes their reactions to the revised OCW.
2.- I evaluated all criteria by filling in the value column.
3.- Place if necessary comments for each criterion.

Range qualification

High	It complies fully
Medium	It complies partially
Low	It doesn´t complies
NA	Not applicable criterion in the site

PRINCIPLE / WCAG 2.0 accessibility guidelines	WCAG 2.0 accessibility criterion	Accessibility level	High	Medium	Low
**PERCEPTIBLE:** The information and components of the user interface must be presented to the user in such a way that they can perceive them.					
**1.1 Text alternatives:** Provide text alternatives for any non–textual content, such as large font, Braille, language, symbols, or simplified language.	1.1.1 Non–textual content: Any non–textual content that is presented should feature alternative text with an equivalent meaning.	**A**			
**1.2 Based on mean time**: Provide alternatives for time–based means of communication.	1.2.1 Audio–only and video– only (pre–recorded): Provide pre–recorded audio and video.	**A**			
	1.2.2 Subtitles (pre–recorded): Provided for all audio present in the content, except when the audio is alternative and is identified as such.	**A**			

	1.2.3 Automatic description of alternative media (pre– recorded): Must be provided for all content, except when it is alternative and is identified as such.	**A**
	1.2.4 Subtitles: Subtitles are provided for all audio or synchronous multimedia content.	**AA**
	1.2.5 Audio description: Must be included in all pre–recorded videos in the multimedia content.	**AA**
	1.2.6 Sign language (pre– recorded): An interpretation of the content in sign language must be provided for all pre–recorded audio in multimedia content.	**AAA**
	1.2.7 Extended audio description (pre–recorded): Where there is a pause in the multimedia content, an extended audio description must be included in all pre– [illegible]	**AAA**
	1.2.8 Multimedia alternative (pre–recorded): A descriptive transcription must be provided for all pre–recorded media.	**AAA**
	1.2.9 Audio only: A descriptive transcription is offered for all direct content that contains only audio.	**AAA**
**1.3 Adaptable:** Create content that can be presented in a variety of ways without losing information or structure	1.3.1 Information and relations: The presented information, structure and relations are programmable or are available in text format.	**A**

	1.3.2 Meaningful sequence: When the sequence of the presentation affects the meaning, the correct sequence can be determined through programming.	**A**
	1.3.3 Sensory characteristics: The instructions provided for the understanding and the content are not based solely on sensory aspects, such as shape, size, visual location, orientation or sound	**A**
1.4 **Distinguishable:** Make it easy for the users to see and listen to content, including the separation from the main background plane.	1.4.1 Use of color: Color must not be the only visual means for information transmission.	**A**
	1.4.2 Audio control: Any audio that is reproduced for longer than 3 seconds must have the option to be paused or disabled.	**A**
	1.4.3 Contrast: The visual presentation of text and text images have a contrast ration of at least 4.5: 1, with the exception of large text, incidental text or banners.	**AA**
	1.4.4 Change the font size: Give the option of changing the font size, except for titles and text images.	**AA**
	1.4.5 Text images: Only in the case of customizable images or particular text presentations.	**AA**
	1.4.6 Contrast (improved): The visual presentation of text and images must have a contrast ration of 7:1, with the exception of large text, incidental text or banners.	**AAA**

	1.4.7 No audio background or with low volume: Pre–recorded audio content is allowed if (1) it is foreground speech, (2) it is CAPTCHA or a banner; and (3) it does not have a [illegible]	**AAA**
	1.4.8 Visual presentation: Understandable visual content, customizable foreground colors, no more than 80 characters per line, justified text, line spacing, font size that can be increased to 200% its original size.	**AAA**
	1.4.9 Text images (no exception): They should not be employed as mere decoration. They must transmit essential information.	**AAA**
**OPERABLE** The user interface and navigation must be operable.		
**2.1 Keyboard Access**: Make every functionality available through the keyboard.	2.1.1 Keyboard: Every functionality must be operable though a keyboard interface.	**A**
	2.1.2 No keyboard traps: If the keyboard can move onto a component in the page, its focus should also be able to exit that component through the keyboard.	**A**
	2.1.3 Keyboard (no exception): Every content functionality can be operated through a keyboard interface, without specific timing of individual keys.	**AAA**
**2.2 Enough Time**: Provide the users enough time to read and use the content.	2.2.1 Adjustable time: For each established time limit in the content.	A
	2.2.2 Pause, stop, hide: For all information that is moving, blinking, being displaced or automatically refreshed.	A

	2.2.3 No timing: The content and functionality must not have time limits, except for multimedia content.	**AAA**	
	2.2.4 Interruptions: Can be postponed or cancelled by the user, with the exception of	**AAA**	
	2.2.5 Re-authentication: The user should be able to re– authenticate their identity and continue their activities without losing any information of the current page if their session expires.	**AAA**	
**2.3 Seizures**: The content should not be designed in a way that may cause seizures.	2.3.1 Three flashes or below threshold: The websites must not contain anything that flashes more than three times in any given one second period or with the flash being under the general flash and red flash thresholds.	**A**	
	2.3.2 Three flashes: The websites do not contain anything that flashes more than three times in any given second.	**AAA**	
**2.4 Navigable**: Provide the users help media for navigation, finding content and determining their location within the website.	2.4.1 Bypass blocks: A mechanism that allows jumping between content blocks that are repeated in multiple pages.	**A**	
	2.4.2 Titled page: Web pages must have titles that describe their topic or purpose.	**A**	
	2.4.3 Focus order: If a web page can be browsed sequentially and the navigation sequence affects its meaning or functionality, there must exist components that maintain the meaning and functionality.	**A**	
	2.4.4 Purpose of a link: The purpose of every link must be identified by its text.	**A**	

	2.4.5 Multiple media: There are multiple ways to locate a web page within a collection of webpages.	**A**	
	2.4.6 Titles and labels: Titles and labels must describe the topic or purpose appropriately.	**AA**	
	2.4.7 Visible focus: Any user interface that can be operated with the keyboard must have a visible indicator of the keyboard focus	**AA**	
	2.4.8 Location: The user is provided with information about their location inside a collection of webpages.	**AAA**	
	2.4.9 Purpose of a link (unique link): There must not exist links with the same text linking to different locations.	**AAA**	
	2.4.10 Section titles: Employed to organize the content.	**AAA**	
**INTELLIGIBLE**<br>The information and functionality of the user interface must be intelligible.			
**3.1 Legible**: The provided content is legible and understandable.	3.1.1 Page language: The language of each page must be programmable or determined by the user.	**A**	
	3.1.2 Language in different parts: The language of each passage or phrase in the content can be selected, with the exception of names, technical terms or undetermined languages.	**AA**	
	3.1.3 Unusual words: Must be defined through adjacent text, a list of definitions, or a glossary.	**AAA**	
	3.1.4 Abbreviations: A mechanism for expanding the meaning of abbreviations must be available.	**AAA**	

	3.1.5 Reading level: Whenever the text requires a more advanced reading level than secondary education, a version that does not require more advanced reading skills must be available.	**AAA**	
	3.1.6 Pronunciation: A specific mechanism must be provided to identify the meanings of words that are ambiguous if their pronunciation is not known.	**AAA**	
	3.2.1 With focus: The context must not change when any component is focused on.	**A**	
**3.2 Predictable**: Present webpages and their operation predictably.	3.2.2 At entry: When any component of the user interface is changed, this does not automatically cause a context change.	**A**	
	3.2.3 Consistent navigation: The browsing mechanisms are repeated in different websites in the same order.	**AA**	
	3.2.4 Consistent identification: Components that have the same functionality within a set of webpages are identified in a coherent manner.	**AA**	
	3.2.5 Changes by request: Context changes must be initiated by user request, and an option should be given to disable such changes.	**AAA**	
**3.3 Input assistance**: Aid the users in avoiding and correcting errors.	3.3.1 Error identification: If a data input error is detected, the item with the error must be automatically identified and described to the user through text.	**A**	

	3.3.2 Instructions or labels: Provided when the content requires the user's intervention.	**A**	
	3.3.3 Suggestions after the error: If an input error is detected, the appropriate suggestions are provided for its correction.	**AA**	
	3.3.4 Error prevention: In law, financial and similar websites, control options must be provided to revert, verify or confirm.	**AA**	
	3.3.5 Help: Context–sensitive help is provided.	**AAA**	
	3.3.6 Error prevention: In websites where the user sends information, options must be provided to revert, verify and confirm.	**AAA**	
**ROBUST**<br>The content must be sufficiently robust to be reliably interpreted by a wide variety of user applications.			
**4.1 Compatible**: maximize compatibility with the current and future user agents, including technical help.	4.1.1 On analysis: The elements have full open and close labels, correctly nested, without duplicated attributes.	**A**	
	4.1.2 Name, role, value: For each user interface component, the name and role can be selected. Their states, properties and values can be established by the user.	**A**	

Thank you for your help
