# Supplementary material for: Accessibility and usability OCW data: The UTPL OCW
Source: Data Brief. 2017 Jun 15;13:582–6. doi: 10.1016/j.dib.2017.06.007 (PMC5496481; doi:10.1016/j.dib.2017.06.007)
Supplement: Supplementary file 7 — Supplementary material [file mmc7.pdf]

Table 8: Questionnaire for the heuristic evaluation of usability

Based on Ma. Suárez Torrente Thesis

**Evaluator:**					
**Date:**		Age:			
**Type of Site:**	Educational / Training	Sex:	M	F	
**URL:**					
**Browser that will be evaluated:**		**Version of the browser**			

Instructions:

1.- For each of the following statements, check the box that best describes their reactions to the revised OCW.

2.- I evaluated all criteria by filling in the value column..

3.- Place if necessary comments for each criterion..

RANGE QUALIFICATION

Value	Description	Value	Description
0	Failure to comply at all	NEP	Failure to follow the main links
10	It complies fully	NPP	Failure to comply on the homepage
NA	Not applicable criterion in the site	NPI	Failure to comply in any interior page
NTS	Failure to comply throughout the site	S	The criterion is met

General Aspects (GA):

Items related to the objectives of the site, the look & feel, consistency and level of content updates.

Code	Criterion	Value
GA.1	Objectives of the web site specific and well defined	
GA.2	Content and services provided accurate and complete	
GA.3	General structure of the web site the user-oriented	
GA.4	Look & Feel general corresponds with the objectives, features, contents and services of the web site	
GA.5	General design of the recognizable web site	
GA.6	General design of the website consistent	
GA.7	Using the language of the user	
GA.8	Support is given to another / s language/s	
GA.9	Complete and correct translation of the website	
GA.10	Web site updated regularly	

Identity and Information (II):

Items related to the identity of the site, the information provided on the supplier and the authorship of the content.

Code	Criterion	Value
II.1	Identity / logo sufficiently visible, identifiable, and significant	
II.2	Identity of the site on all pages	
II.3	Slogan or tagline appropriate to the objective of the site	
II.4	Provides company information on the web site	
II.5	There are contact mechanisms	
II.6	Does site offer information about the protection of personal rights of the author or web site.	
II.7	Provides information about the author, sources and dates of creation and revision of articles, news and reports	

Structure and navigation (SN):

Items related to the suitability of the information architecture and navigation of the site.

Code	Criterion	Value
SN.1	It has avoided welcome screen	
SN.2	Structure of organization and navigation adequate	
SN.3	Organization of elements consistent with the conventions	
SN.4	Control of the number of elements and of terms by element in the navigation menu	
SN.5	Balance between depth and width in the case of hierarchical structure	
SN.6	Easily recognizable links	
SN.7	The characterization of the links indicates their status (active, visited)	
SN.8	There is no redundancy of links	
SN.9	No broken links	
SN.10	There are no links that lead to the same page displayed	
SN.11	Link images indicates the content to be accessed	
SN.12	There is a link to return to the home on every page	
SN.13	There are elements of navigation that guide the user about where it is and how to dispose your navigation (eg: crumbs)	
SN.14	There is a site map to directly access the content without browsing	

Labelled (LB):

Elements related with the significance, correction and familiarity of the labeled of the contents.

Code	Criterion	Value
LB.1	Significant signs	
LB.2	Labeled system controlled and accurate	
LB.3	The title pages, correct and planned	
LB.4	Home › correct, clear, and easy to remember URL	
LB.5	Clear Web pages and URLs	
LB.6	URLs of pages internal and permanent	

Layaout of the page (LY):

Items related to the distribution and the appearance of the elements of navigation and information in the interface.

Code	Criterion	Value
LY.1	Take advantage of the zones of high content hierarchy for more relevant information	
LY.2	We have avoided the information overload	
LY.3	no visual noise, and a clean interface	
LY.4	There are areas in white between objects on the information page to be able to view the rest	
LY.5	Use correct visual spacing of the page	
LY.6	Used correctly the visual hierarchy to express the relations of the type "part of" between elements on the page	
LY.7	control the length of the page	
LY.8	The printed version of the page is correct	
LY.9	The text of the page is read without difficulty	
LY.10	Avoid blinking text / slider	

Comprehensibility and ease of Interaction (CI):

Items related to the adequacy and quality of text content, icons and interface controls.

Code	Criterion	Value
CI.1	Use clear and concise language	

CI.2	Family and friendly language	
CI.3	Each paragraph expresses an idea	
CI.4	Consistent use of the interface controls	
CI.5	Metaphors Visual recognizable and understandable for any user (eg.: icons)	
CI.6	Using drop-down menus, coherent or alphabetical order	
CI.7	If the user has that fill a field, the options available can select instead of having that write them	

Control and feedback (CF):

Items related to freedom of the user navigation and the information provided to it in the process of interaction with the site.

Code	Criterion	Value
CF.1	The user has full control over the interface	
CF.2	Are reported to the user about what is happening	
CF.3	The user is informed of what has happened	
CF.4	There are validation systems before the user submits information to prevent errors	
CF.5	When it produces an error, it reports to the user what happened and of how solve the problem	
CF.6	It has controlled the time of response	
CF.7	We have avoided the site windows to override or to overlap the browser	
CF.8	We have avoided the proliferation of windows on the screen of the user	
CF.9	It has avoided the download from the user's plugins	
CF.10	If there are tasks of several steps, it indicates to the user which it is and how many are missing to complete the task	

Multimedia elements (ME):

Elements related to the degree of adequacy contained in the multimedia web site.

Code	Criterion	Value
ME.1	Well cropped pictures	
ME.2	Understandable photographs	
ME.3	Photographs with correct resolution	
ME.4	The use of images or animations provides some type of value added	
ME.5	We have avoided the use of cyclic animations	
ME.6	The use of sound provides some sort of added value	

Search (SE):

The search engine implemented on web site-related items.

Code	Criterion	Value
SE.1	The search, if is necessary, is is accessible from all the pages of the site	
SE.2	It is easily recognizable as such	
SE.3	It is easily accessible	
SE.4	The text box is wide enough	
SE.5	Simple and clear search system	
SE.6	It allows advanced search	
SE.7	Displays the search results in understandable way for the user	
SE.8	It assists the user in case of not offering results for a given command	

Help (HE):

Elements related with the support offered to the user during the navigation for the site.

Code	Criterion	Value
AY.1	The link to the help section is placed in a visible area	

AY.2 Easy access and return to the / help system
AY.3 It offers help in complex and contextual tasks
AY.4 FAQs (if there are any) correct choice as to the writing of the questions
AY.5 FAQs (if any) correct the wording of the responses

THANK YOU VERY MUCH FOR YOUR HELP!
